# Relationship between body composition and bone mass in normal-weight and overweight adolescents

**DOI:** 10.7717/peerj.14108

**Published:** 2022-11-03

**Authors:** Mateus Augusto Bim, André de Araujo Pinto, Hector Cris Colares de Angelo, Isadora Gonzaga, Adriana Coutinho de Azevedo Guimarães, Érico Pereira Gomes Felden, Wellington Roberto Gomes de Carvalho, Karen Hind, Andreia Pelegrini

**Affiliations:** 1 Universidade do Estado de Santa Catarina, Florianópolis, Brazil; 2 Universidade Estadual de Roraima, Boa Vista, Brazil; 3 Universidade Federal do Triângulo Mineiro, Uberaba, Brazil; 4 Durham University, Durham, United Kingdom

**Keywords:** Adolescent, Bone mineral density, Bone mineral content, X-ray absorptiometry, Body weight

## Abstract

Adolescence is a period characterized by large accumulation of bone mass. Body composition is an important determinant of bone mass. This study aimed to assess the relationship of bone mass with lean mass (LM) and fat mass (FM) in normal-weight and overweight adolescents with consideration of sex, sexual maturation and physical activity covariates. A total of 118 adolescents (60 girls and 58 boys) aged between 10 and 14 years participated in the study. Individuals were classified as normal weight or overweight according to body mass index. Bone mineral density (BMD), bone mineral content (BMC), LM, and FM were measured by dual-energy X-ray absorptiometry. In normal-weight adolescents, LM (*β* = 0.725, *p* < 0.001) and FM (*β* = 0.185, *p* = 0.019) were associated with lumbar spine BMC, whereas in overweight adolescents only LM (*β* = 0.736, *p* < 0.001) was associated with lumbar spine BMC. Furthermore, in the normal-weight group, FM and LM were associated with total body less head BMD (LM, *β* = 0.792, *p* < 0.001; FM, *β* = 0.257, *p* = 0.007) and lumbar spine BMD (LM, *β* = 0.553, *p* < 0.001; FM, *β* = 0.199, *p* < 0.035). In the overweight group, only LM was associated with total body less head BMD (*β* = 0.682, *p* < 0.001) and lumbar spine BMD (*β* = 0.528, *p* < 0.001). LM was the main predictor of bone mass in normal-weight and overweight adolescents. FM was associated with bone mass in normal-weight adolescents only. LM may be considered an important and useful marker in adolescents, when investigating bone health in this population. Activities that promote LM gain to reduce the risk of bone fractures and diseases in adulthood are recommended.

## Introduction

Osteoporosis, a metabolic disease affecting millions of people worldwide, is known to result from an imbalance between osteoclastic bone resorption and osteoblastic bone formation (Torres-Costoso et al., 2020; Zhu & Zheng, 2021). An important risk factor for osteoporosis development is low peak bone mass, defined as a low amount of accumulated bone mass at the end of skeletal maturity (Zhu & Zheng, 2021). Adolescence is a crucial period for the development of bone mass, which increases by about 45% during puberty, reaching 90% of the peak bone mass of an adult individual (Bland et al., 2020a; Bland et al., 2020b). As such, it is essential to enhance bone mass accumulation during adolescence in order to prevent later osteoporosis.

In adolescents, body composition, a parameter combining fat mass (FM) and lean mass (LM), has been associated with bone mineral density (BMD) (Jeddi et al., 2015; Sioen et al., 2016; Han, Kim & Kim, 2021), a measurement of the mineral mass of bone per unit area, expressed in g/cm2 (International Atomic Energy Agency, 2010). LM shows a clear association with bone health, explained by the mechanical loading transferred to the skeleton (Deng et al., 2021). The effect of FM on bone health, however, is controversial, with reports of positive and negative associations in studies with adolescents (Deng et al., 2021) and adults (Hamrick, 2011). On the one hand, the mechanical loading of extra mass may be favorable to bone development, but on the other hand, high levels of fat mass can lead to inflammation and oxidative stress, culminating in negative effects (Hamrick, 2011; Shapses, Pop & Wang, 2017).

Associations between weight classification (underweight, normal weight, overweight and obese) and bone health have been increasingly studied in adolescents (van Leeuwen et al., 2017). While minimal difference between overweight and obese classifications have been documented regarding bone mineral content (BMC), the mineral mass component of bone measured in grams (International Atomic Energy Agency, 2010), differences in BMD were significant between these weight groups, with higher BMD values for obese (van Leeuwen et al., 2017).

Furthermore, this difference in BMD between weight groups tends to be accentuated when obese and/or overweight adolescents are compared to those with normal weight (van Leeuwen et al., 2017). These findings underscore the need for studies examining the relationship of LM and FM with bone health according to weight group, given that such relationships may be group-specific.

For a better understanding of the relationship between body composition and bone mass in adolescents and potential implications for later life stages, it is important to determine which body composition components best explain bone mass parameters in this pediatric population. It is necessary to elucidate to which extent LM and FM are related, individually, to bone mass and whether weight classification implies differences in these relationships. Considering these issues, this study aimed to evaluate the relationship between bone mass and FM and LM in normal weight and overweight adolescents.

## Materials & Methods

### Study sample

This cross-sectional study is part of a research project entitled “Bone mineral density in adolescents: What is its relationship with body fat, physical activity, and sedentary behavior?”, conducted in Sao José, SC, Brazil, from April to December 2016. The project was approved by the Human Research Ethics Committee at Santa Catarina State University (protocol No. 1,468,045/2016). The target population was male and female adolescents aged between 10.0 and 14 years enrolled in a primary school in Sao José, SC, Brazil. Participants were selected from a list, provided by the school, containing body mass index (BMI) values collected in the previous year. The exclusion criteria were fracture history, malignant neoplasms, chromosomal abnormalities, paralysis, renal and hepatic insufficiency, hyper- and hypothyroidism, chronic viral infections, such as human papillomavirus (HPV), human immunodeficiency virus (HIV), human T-lymphotropic virus (HTLV), and any other disease that could interfere with body composition. All participants and their parents/guardians provided informed written consent to participate in the study.

**Figure 1:**
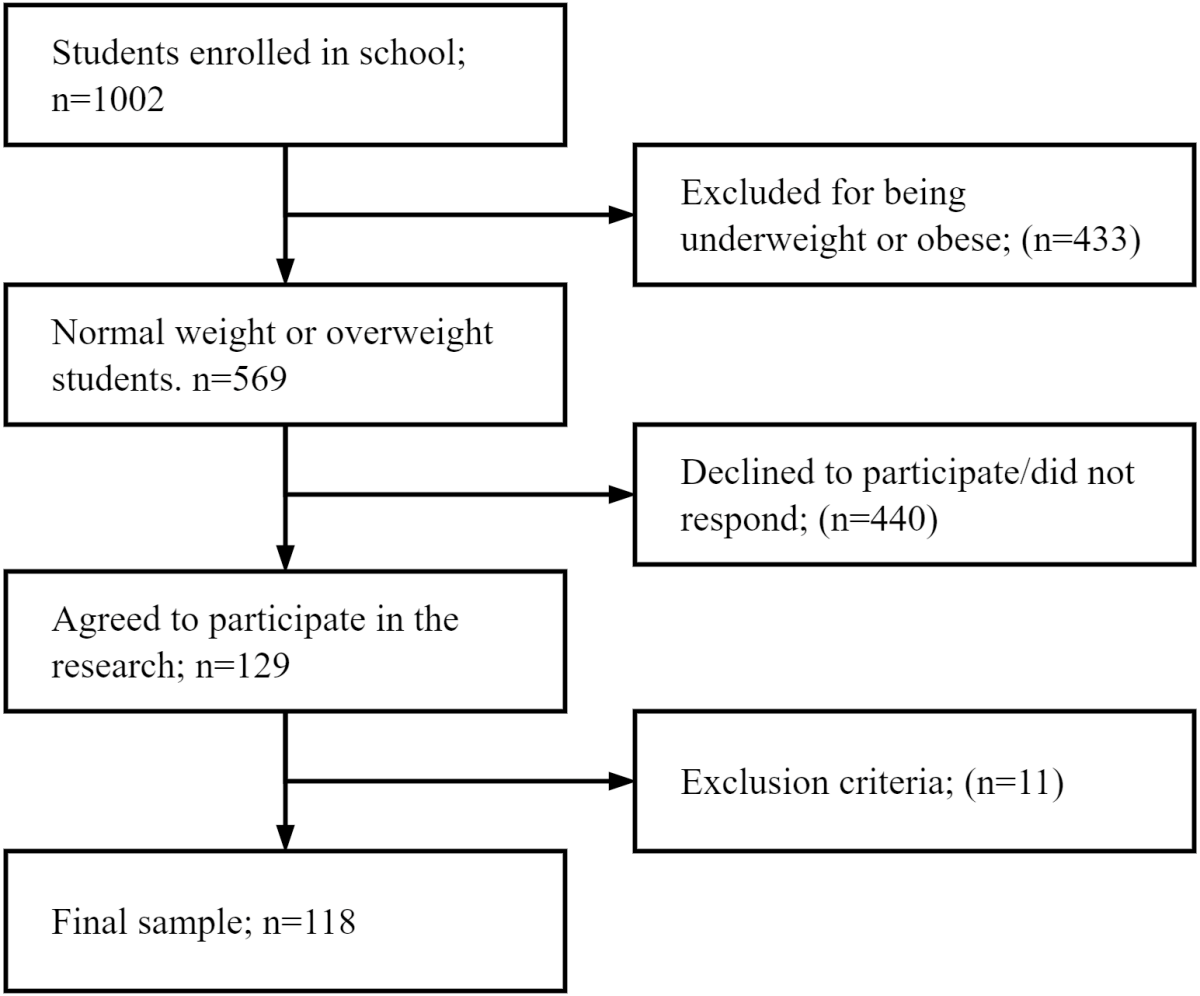
Flowchart of the sample selection.

The total sample consisted of 118 adolescents (60 girls and 58 boys) who were grouped according to confirmed BMI: normal weight and (34 girls and 32 boys) overweight (26 girls and 26 boys), according to sex and age (Cole et al., 2000; Cole et al., 2007) (Fig. 1).

This sample was deemed adequate for the study according to the sample size power calculation (G*Power, version 3.1.9.4). *R*^2^ values of the multiple linear regression model for total body less head (TBLH) BMD (normal weight = 0.571, overweight = 0.502) were used for calculations, affording statistical power values of 1.00 and 0.99 for the normal-weight and overweight groups, respectively.

Data collection was performed in two stages. The first stage was carried out at school to confirm participant BMI. The second stage was conducted at the Laboratory of Anthropometry, Health Sciences Center, Santa Catarina State University. Prior to data collection, students were instructed to (i) observe an overnight fast or fast for ∼10 h before their scheduled appointment, (ii) wear lightweight and comfortable clothing, (iii) not wear any metal objects, such as earrings, necklace, or watch, (iv) not exercise up to 8 h before measurements, (v) bladder voided, and (vi) not drink alcohol up to 48 h prior (Nana et al., 2015).

### Anthropometry

Body mass (kg) and height (m^2^) measurements were obtained according to recommendations of the International Society for the Advancement of Kinanthropometry (Canadian Society for Exercise Physiology , CSEP). Body weight was measured on a digital scale (G Tech Pro^®^, Pacific Palisades, USA) with a maximum capacity of 150 kg and a resolution of 100 g. Height was measured by using a stadiometer (Sanny^®^, Sao Paulo, Brazil) with a resolution of 0.1 cm. Body weight and height data were used to calculate BMI (Cole et al., 2000; Cole et al., 2007). BMI was collected in the first stage of the study to select normal-weight and overweight students who were recruited for the second stage of the study.

### Dual energy X-ray absorptiometry

Bone parameters and body composition were estimated using whole body dual-energy X-ray absorptiometry (DXA) on a Hologic system (Discovery Wi Fan-Beam; Bedford, Massachusetts, USA). In accordance with the procedures recommended by the manufacturer, the equipment was calibrated daily and weekly. The same laboratory technician led positioning for each scan, performed the examinations and analysis according to the operator’s manual using the standard analysis protocol. DXA provided data on BMC (g), BMD (g/cm^2^), LM (kg), and FM (kg). The pediatric software (Hologic Auto Whole Body, version 12.4.5) was used to calculate TBLH and lumbar spine (L2–L4) BMC and BMD.

### Covariates

Maturation was self-reported following the criteria proposed by Tanner (1962) and adapted by Adami & Vasconcelos (2008). The adolescents received instructions individually from a research team member in a private environment. A self-assessment was performed using a worksheet with figures corresponding to the five stages of pubertal development. Pubic hair development (PH1–PH5) was observed for boys and girls. Physical activity level was estimated using a triaxial accelerometer (Actigraph GT3XE-Plus) that measured acceleration produced by movement. Adolescents were instructed to affix the accelerometer on the right half of the body in the transverse line of the waist, above the iliac crest, by using an elastic belt. They were advised to maintain the accelerometer attached to the body during all times from waking up to going to bed for 10 consecutive days, and only remove it during water-based activities, such as swimming. Data, expressed in minutes of total physical activity per day, were analyzed using ActiLife software. For data analysis, a sample interval (epoch) of 15 s, three valid weekdays and two valid weekend days (minimum of 10 h of accelerometer use per day) were used. Periods of 20 consecutive zeros of activity with a tolerance of 2 min were excluded. The first and last days of accelerometer use, as well as days with less than 600 min of recordings, were excluded from analysis. Physical activity was treated as the sum of moderate and vigorous physical activity.

### Statistical analysis

Data were entered into EpiData 3.1 (EpiData Association, Odense, Denmark) by two researchers using an automated data checking tool. Later, the data were exported to IBM SPSS Statistics version 20.0 for cleaning and analysis. Descriptive statistics (mean, standard deviation, and frequency distribution) were calculated. Data normality was assessed by the Kolmogorov–Smirnov test. Variables that did not follow a normal distribution were transformed to log_10_ values. The independent samples *t*-test and the Mann–Whitney *U*-test were used for comparison of means. The chi-square test was used to investigate the association between sexual maturation and weight classification. For association analyses, multiple linear regression (enter method) was performed, and models were adjusted for sex, sexual maturation, and physical activity. All analyses were conducted using a 95% confidence level (*p* < 0, 05). Data are available on the OSF repository (DOI http://dx.doi.org/10.17605/OSF.IO/GKDA4).

## Results

Boys showed higher moderate-to-vigorous physical activity and LM, whereas girls had higher means for FM, TBLH BMC, lumbar spine BMC, and lumbar spine BMD (Table 1). In comparing normal-weight and overweight adolescents, overweight adolescents had higher body weight, height, BMI, FM, LM, lumbar spine BMC, TBLH BMD, and lumbar spine BMD. The frequency of normal-weight adolescents was higher at maturation stages 1, 2, and 3 and that of overweight adolescents was higher at stages 4 and 5 (Table 2).

**Table 1:** Study sample descriptive data by sex.

	Total sample, *n* = 118	Male, *n* = 58	Female *n* = 60	Difference *p*
	$\bar {x}$ (sd)	$\bar {x}$ (sd)	$\bar {x}$ (sd)	
Age (years)	12.2 (1.2)	12.2 (1.2)	12.2 (1.1)	0.756^b^
Weight (kg)	49.7 (12.0)	49.3 (12.0)	50.2 (12.1)	0.695^a^
Height (cm)	156.5 (9.8)	156.8 (10.8)	156.2 (8.8)	0.716^a^
BMI (kg/m^2^)	20.1 (3.6)	19.9 (3.4)	20.4 (3.8)	0.357^b^
MVPA (min/day)	40.7 (22.6)	50.0 (27.2)	31.7 (11.5)	<0.001^a^
FM (kg)	14.8 (7.5)	13.1 (7.0)	16.4 (7.7)	0.016^b^
LM (kg)	29.3 (6.4)	30.9 (7.4)	27.7 (5.0)	0.007^a^
BMC (g)				
*TBLH*	1478.038 (486.358)	1283.033 (351.324)	1666.543 (525.657)	<0.001^a^
*Lumbar spine*	40.476 (15.314)	36.043 (12.361)	44.762 (16.717)	0.002^a^
BMD (g/cm^2^)				
*TBLH*	0.903 (0.113)	0.885 (0.091)	0.919 (0.129)	0.099^a^
*Lumbar spine*	0.920 (0.191)	0.831 (0.131)	1.006 (0.202)	<0.001^a^
	*n* (%)	*n* (%)	*n* (%)	
Sexual maturation				0.294
Stage 1	10 (8.5)	6 (10.3)	4 (6.7)	
Stage 2	25 (21.2)	11 (19.0)	14 (23.3)	
Stage 3	29 (24.6)	10 (17.2)	19 (31.7)	
Stage 4	48 (40.7)	27 (46.6)	21 (35.0)	
Stage 5	6 (5.1)	4 (6.9)	2 (3.3)	

**Notes.**

$\bar {x}$ mean sd standard deviation BMI body mass index MVPA moderate-to-vigorous physical activity FM fat mass LM lean mass BMD bone mineral density TBLH total body less head BMC bone mineral content

a Independent*T* test.

b Mann–Whitney*U* test.

**Table 2:** Study sample descriptive data by weight category.

	Normal weight, *n* = 66$\bar {x}$ (sd)	Overweight, *n* = 52$\bar {x}$ (sd)	Difference *p*
Age (years)	12.2 (1.3)	12.2 (1.1)	0.754^b^
Weight (kg)	41.8 (7.3)	59.8 (9.0)	<0.001^a^
Height (cm)	154.4 (9.4)	159.1 (9.8)	0.009^a^
BMI (kg/m^2^)	17.5 (2.1)	23.5 (1.7)	<0.001^a^
MVPA (min/day)	41.3 (27.2)	39.9 (15.1)	0.733^a^
FM (kg)	9.7 (4.6)	21.2 (5.1)	<0.001^a^
LM (kg)	26.9 (5.1)	32.3 (6.8)	<0.001^a^
BMC (g)			
TBLH	1529.253 (515.103)	1413.035 (443.611)	0.199^a^
Lumbar spine	35.848 (11.021)	46.350 (17.898)	<0.001^b^
BMD (g/cm^2^)			
TBLH	0.860 (0.077)	0.957 (0.129)	<0.001^a^
Lumbar spine	0.863 (0.161)	0.993 (0.203)	<0.001^b^
	*n* (%)	*n* (%)	
Sexual maturation			0.036
Stage 1	7 (10.6)	3 (5.8)	
Stage 2	17 (25.8)	8 (15.4)	
Stage 3	17 (25.8)	12 (23.1)	
Stage 4	25 (37.9)	23 (44.2)	
Stage 5	0 (0.0)	6 (11.5)	

**Notes.**

$\bar {x}$ mean sd standard deviation BMI body mass index MVPA moderate-to-vigorous physical activity FM fat mass LM lean mass BMD bone mineral density TBLH total body less head BMC bone mineral content

a Independent*T* test.

b Mann–Whitney*U* test.

Table 3 present the multiple linear regression models for the associations of BMC and BMD with body composition, with adjustment for sex, sexual maturation and physical activity. No association was observed between TBLH BMC and FM or LM in either group. In normal-weight adolescents, LM and FM were predictors of lumbar spine BMC, which explained 69.6% of the variance in the outcome (*F* (5, 60) = 27.425, *p* < 0.001; *R*^2^ = 0.696). On the other hand, in overweight adolescents, only LM explained the variance in lumbar spine BMC (*F* (5, 46) = 11.382, *p* < 0.001; *R*^2^ = 0.553). Regarding TBLH BMD, in normal-weight adolescents, LM and FM were associated with the variable, explaining 57.1% of the variance in TBLH BMD (*F* (5, 60) = 15.951, *p* < 0.001; *R*^2^ = 0.571). In overweight adolescents, only LM was associated with TBLH BMD, explaining 50.2% of the variance in the outcome (*F* (5, 46) = 9.291, *p* < 0.001; *R*^2^ = 0.502). LM and FM were significantly associated with lumbar spine BMD in normal-weight adolescents, explaining 56.4% of the variance in the variable (*F* (5, 60) = 15.517, *p* < 0.001; *R*^2^ = 0.564). In overweight adolescents, only LM was associated with lumbar spine BMD, explaining 58.9% of the variance in the variable (*F* (5, 46) = 13.208, *p* < 0,001; *R*^2^ = 0.589) (Table 3).

**Table 3:** Multiple linear regression models for associations between bone mineral content, bone mineral density and independent variables, stratified by weight status (normal weight and overweight).

	Normal weight (*n* = 66)^a^				Overweight (*n* = 52)^b^			
	B ± SE	**(B**_95%*CI*_)	*β*	**t**	B ± SE	(B_95%*CI*_)	*β*	**t**
**BMC TBLH**								
Constant	996.491 ± 375.614	245.151–1747.831		2.653	1395.197 ± 414.576	560.698–2229.696		3.365
LM	30.926 ± 17.097	−3.273–65.124	0.307	1.809	−8.220 ± 11.189	−30.743–14.303	−0.125	−0.735
FM	−21.674 ± 13.282	−48.242–4.894	−0.192	−1.632	19.239 ± 13.343	−7.619–46.098	0.221	1.442
**BMC Lumbar spine**								
Constant	−15.889 ± 5.246	−26.382 to −5.397		−3.029	−23.756 ± 12.519	−48.956–1.444		−1.898
LM	1.563 ± 0.239	1.086–2.041	0.725^*^	6.547	1.947 ± 0.338	1.267–2.628	0.736^*^	5.763
FM	0.446 ± 0.185	0.075–0.817	0.185^**^	2.406	0.087 ± 0.403	−0.724–0.898	0.025	0.215
**BMD TBLH**								
Constant	0.505 ± 0.044	0.417–0.592		11.566	0.440 ± 0,095	0.249–0.631		4.635
LM	0.012 ± 0.002	0.008–0.016	0.792^*^	6.020	0.013 ± 0,003	0.008–0.018	0.682^*^	5.066
FM	0.004 ± 0.002	0.001–0.007	0.257^**^	2.806	0.002 ± 0,003	−0.005–0.008	0.061	0.499
**BMD Lumbar spine**								
Constant	0.255 ± 0.092	0.071–0.439		2.778	0.228 ± 0.136	−0.047–0.502		1.671
LM	0.017 ± 0.004	0.009–0.026	0.553^*^	4.173	0.016 ± 0.004	0.008–0.023	0.528^*^	4.315
FM	0.007 ± 0.003	0.001–0.014	0.199^**^	2.159	0.003 ± 0.004	−0.006–0.012	0.078	0.705

**Notes.**

LM lean mass FM fat mass B unstandardized coefficient SE standard error *β* standardized coefficient BMC bone mineral content BMD bone mineral density TBLH total body less head

a *R*^2^ = 0.286 for TBLH BMC; *R*^2^= 0.696 for lumbar spine BMC.

b *R*^2^ = 0.202 for TBLH BMC; *R*^2^= 0.553 for lumbar spine BMC.

a *R*^2^= 0.571 for TBLH BMD; *R*^2^ = 0.564 for lumbar spine BMD.

b *R*^2^ = 0.502 for TBLH BMD; *R*^2^ = 0.589 for lumbar spine BMD.

Models were adjusted for sex, sexual maturation, and moderate-to-vigorous physical activity.

## Discussion

The main finding from this study was that LM was the best predictor of bone mass in normal-weight and overweight adolescents, after adjustment for possible confounders (*i.e.*, sex, sexual maturation, physical activity). In normal-weight adolescents, both LM and FM were associated with lumbar spine BMD, TBLH BMD, and lumbar spine BMC, with LM explaining the greatest variance in bone variables. In overweight adolescents, only LM was associated with lumbar spine BMD, TBLH BMD, and lumbar spine BMC. Overweight adolescents had higher lumbar spine BMC, TBLH BMC, and lumbar spine BMD than normal-weight adolescents.

The higher BMC and BMD values in overweight adolescents compared with normal-weight adolescents corroborate the results of a systematic review (van Leeuwen et al., 2017). Such findings might be associated with mechanical loading applied on the skeleton by increased body weight (van Leeuwen et al., 2017). However, after adjusting for body size, previous studies have found that the bone mass of obese children might be lower than that of normal-weight children (Dimitri et al., 2012; Dimitri, 2019). This has also been reported in obese adults who have lower BMD relative to body mass (Rudman et al., 2019; Wang et al., 2020). Furthermore, adipose tissues may exert adverse effects on bone health, resulting from inflammation, oxidative stress, and derivation of adipocytes and osteoblasts from mesenchymal stem cell progenitors (Shapses, Pop & Wang, 2017). These findings reinforce that overweight/obesity, in addition to increasing the risk for developing other diseases, may negatively impact bone health.

It should be noted that studies comparing bone outcomes in adolescents with different weight classification (*e.g.*, normal weight, overweight, or obese) do not always consider LM or FM distribution. This may lead to the understanding that higher FM and LM values may enhance bone mass in overweight adolescents. However, the findings of the present study showed that only LM was associated with BMD (lumbar spine and TBLH) and BMC (lumbar spine) in overweight adolescents. An increased amount of LM may enhance the mechanical load and generate greater muscle tension on bones, stimulating bone development (Torres-Costoso et al., 2020; Saraiva et al., 2021). These results may also reflect the greater engagement of adolescents in physical activities, since the practice of moderate-to-vigorous physical activities intensities has a positive relationship with bone parameters (Bland et al., 2020a; Bland et al., 2020b). Zymbal et al. (2019) identified a mediating effect of LM on the association between physical activity and bone mineral density, indicating that the practice of physical activity promotes an increase in muscle mass, due to the increase in mechanical loads applied to the muscles, resulting in beneficial adaptations to the skeleton.

The association of FM with bone mass in children and adolescents is inconsistent (Sioen et al., 2016). In the present study, FM was associated with lumbar spine BMD, TBLH BMD, and lumbar spine BMC only in normal-weight adolescents. The relationship between fat mass and bone may in part be explained through the positive influence of leptin, produced by adipose tissue, on bone formation (Reid, 2008). In overweight adolescents, by contrast, FM was not associated with BMC or BMD. Overweight adolescents had greater FM content than normal-weight adolescents. Unlike in normal-weight adolescents, in which FM was found to positively influence bone mass, in overweight individuals, excess FM may not provide such benefits and we suggest that there may be a threshold of FM whereby any further accumulation might be negative for bone. Although our study did not include obese individuals, research has shown that obese children and adolescents are 25% more likely to suffer fractures of the extremities than non-obese children and adolescents (Kim et al., 2016), which may be indicative of obesity-induced skeletal fragility.

The scientific literature has revealed that low-grade chronic inflammation can negatively impact bone health in individuals with excess FM (Faienza et al., 2019; Gkastaris et al., 2020). This is due to upregulation of pro-inflammatory cytokines (*e.g.*, interleukin-6 and tumor necrosis factor-alpha), which positively regulate osteoclast activity and inhibit osteoblastogenesis, resulting in increased bone resorption (Gkastaris et al., 2020). When adjusted for LM, the association between FM and bone mass may be reduced or even non-significant, suggesting that LM is the main predictor of bone mass in overweight and obese individuals (Sioen et al., 2016).

In normal-weight adolescents, both FM and LM were associated with lumbar spine BMD, TBLH BMD, and lumbar spine BMC. Jeddi et al. (2015) reported that LM was the most important predictor of BMD in adolescents of both sexes. Such findings might be explained by the hormonal effects related to increased conversion of androstenedione to estrogen and the high circulating level of leptin, as leptin receptors are mediated by muscles (Olmedillas et al., 2010).

One of the limitations of this study was the small sample size, which precluded stratification by sex. Furthermore, there was no control over other variables that may influence bone development (*e.g.*, levels of circulating hormones that act in the calcification process, nutritional data, adequate intake and production of calcium and vitamin D, sun exposure, mechanical overload imposed on the skeleton, bone remodeling biomarkers, pro-inflammatory cytokines), although adolescents with conditions known to adversely affect bone health, were excluded from the study. As strengths of the study, we highlight the use of DXA, for simultaneously measuring bone density and important indices of body composition in children. Future research should focus on population cohorts and include underweight and obese adolescents to enable comparisons with normal-weight and overweight. It is also pertinent to investigate the relationship between different body fat compartments, such as visceral fat, and bone mass.

## Conclusions

In conclusion, LM was the main predictor of BMC and BMD in normal-weight and overweight adolescents, whereas FM was positively associated with BMD in normal-weight adolescents only. LM may be considered an important and useful marker in adolescents, when investigating bone health in this population. Such evidence is important for providing recommendations on activities that promote LM gain to reduce the risk of bone fractures and diseases in adulthood.

##  Supplemental Information

10.7717/peerj.14108/supp-1 Supplemental Information 1 Raw Data

## Additional Information and Declarations

### Data Availability

The following information was supplied regarding data availability: The data are available at OSF: Bim, Mateus A. 2022. “Relationship between Body Composition and Bone Mass in Adolescents with Normal Weight and Overweight.” OSF. May 31. DOI: 10.17605/OSF.IO/GKDA4.
